# Glibenclamide Decreases ATP-Induced Intracellular Calcium Transient Elevation via Inhibiting Reactive Oxygen Species and Mitochondrial Activity in Macrophages

**DOI:** 10.1371/journal.pone.0089083

**Published:** 2014-02-18

**Authors:** Duo-ling Li, Zhi-yong Ma, Zhi-jie Fu, Ming-ying Ling, Chuan-zhu Yan, Yun Zhang

**Affiliations:** 1 Key Laboratory of Cardiovascular Remodeling and Function Research, Chinese Ministry of Education and Chinese Ministry of Public Health, Qilu Hospital, Shandong University, Jinan, China; 2 Department of Neurology, Qilu Hospital, Shandong University, Jinan, China; 3 Department of Otorhinolaryngology, Shandong Provincial Qianfoshan Hospital, Shandong University, Jinan, China; Temple University, United States of America

## Abstract

Increasing evidence has revealed that glibenclamide has a wide range of anti-inflammatory effects. However, it is unclear whether glibenclamide can affect the resting and adenosine triphosphate (ATP)-induced intracellular calcium ([Ca^2+^]_i_) handling in Raw 264.7 macrophages. In the present study, [Ca^2+^]_i_ transient, reactive oxygen species (ROS) and mitochondrial activity were measured by the high-speed TILLvisION digital imaging system using the indicators of Fura 2-am, DCFDA and rhodamine-123, respectively. We found that glibenclamide, pinacidil and other unselective K^+^ channel blockers had no effect on the resting [Ca^2+^]_i_ of Raw 264.7 cells. Extracellular ATP (100 µM) induced [Ca^2+^]_i_ transient elevation independent of extracellular Ca^2+^. The transient elevation was inhibited by an ROS scavenger (tiron) and mitochondria inhibitor (rotenone). Glibenclamide and 5-hydroxydecanoate (5-HD) also decreased ATP-induced [Ca^2+^]_i_ transient elevation, but pinacidil and other unselective K^+^ channel blockers had no effect. Glibenclamide also decreased the peak of [Ca^2+^]_i_ transient induced by extracellular thapsigargin (Tg, 1 µM). Furthermore, glibenclamide decreased intracellular ROS and mitochondrial activity. When pretreated with tiron and rotenone, glibenclamide could not decrease ATP, and Tg induced maximal [Ca^2+^]_i_ transient further. We conclude that glibenclamide may inhibit ATP-induced [Ca^2+^]_i_ transient elevation by blocking mitochondria K_ATP_ channels, resulting in decreased ROS generation and mitochondrial activity in Raw 264.7 macrophages.

## Introduction

Glibenclamide is widely used to treat type 2 diabetes [1]. The pharmacological action of glibenclamide is to inhibit adenosine triphosphate (ATP)-sensitive K^+^ channels (K_ATP_) in pancreatic β cells, leading to the stimulation of insulin secretion [2]. Meanwhile, increasing evidence has revealed that glibenclamide also has a wide range of anti-inflammatory effects [3], [4]. Recently, we found that glibenclamide could ameliorate the progression of atherosclerosis and reduce the production of inflammatory cytokines as well as the phosphorylation of p65 and ERK1/2 in Raw 264.7 macrophages [5]. However, the mechanism responsible for the anti-inflammatory effect of glibenclamide is largely unexplored.

Previous studies have found that Ca^2+^ plays a critical role in the biochemical cascade of signal transduction pathways, resulting in the activation of immune cells [6], [7]. Because glibenclamide was found to increase the intracellular Ca^2+^ concentration ([Ca^2+^]_i_) in pancreatic β cells [2], investigating whether glibenclamide was able to affect [Ca^2+^]_i_ in Raw 264.7 macrophages was considered worthwhile.

As the main effector cells at sites of inflammation and tissue injury, macrophages are likely to be exposed to many extracellular molecules that are involved in cellular signaling [8], [9]. In particular, extracellular ATP was found to be one of the key molecules in modulating the immune response through their capacity to bind and activate multiple nucleotide receptor family members [10]. In non-excitable cells, extracellular ATP induces an elevation of cytosolic calcium by two distinct mechanisms, either by the activation of Ca^2+^ release from intracellular Ca^2+^ stores or by the activation of Ca^2+^ influx from the extracellular medium [11], [12]. However, it is unclear whether glibenclamide has any effect on ATP-induced [Ca^2+^]_i_ handling.

Additionally, previous studies found that there was cross-talk between [Ca^2+^]_i_ and intracellular reactive oxygen species ([ROS]_i_) signaling generated from mitochondria [13], [14]. As we know, glibenclamide can block mitochondrial K_ATP_ channels, which play an important role in [ROS]_i_ production [15]. Therefore, we hypothesized that [ROS]_i_, mainly from mitochondria, participated in the regulation of ATP-induced [Ca^2+^]_i_ transient elevation and that glibenclamide might inhibit the [Ca^2+^]_i_ transient elevation by inhibiting ROS generation and blocking mitochondrial K_ATP_ channels.

## Materials and Methods

### Cell culture

Murine macrophage cell line Raw 264.7 cells (American Type Culture Collection, Manassas, VA) were cultured in DMEM supplemented with 10% fetal calf serum, 100 µg/ml streptomycin and 100 U/ml penicillin at 37°C and in 5% CO_2_ and 95% air.

### Intracellular calcium measurements

Calcium imaging was performed as we described previously [16]. Briefly, Raw 264.7 cells were incubated with 2 µM fura-2/acetoxymethylester for 30 min at 37°C and then were washed out at room temperature for another 30 min. Measurements were made using an inverted microscope (Nikon TE2000-U, Nikon, Japan) and a TILLvisION digital imaging system (TILL Photonics GmbH, Munich, Germany) as reported previously [16]. [Ca^2+^]_i_ was indicated as the ratio of fluorescence intensity at excitation wavelengths of 340 and 380 nm (F ratio). The emission wavelength was 510 nm. The background intensity was subtracted from the fluorescent intensity changes, and the resulting [Ca^2+^]_i_ values were normalized as the differences between the fluorescence intensities with different agents and the intensity in standard bath solution (BS) by averaging the values of at least 50 cells/treatment.

### Measurement of intracellular ROS levels

The production of intracellular ROS was monitored by dichlorodihydrofluorescein diacetate (H_2_DCFDA) as a fluorescent dye. The cells were trypsinized, and the cell suspension was treated with H_2_DCFDA at a final concentration of 10 µM in the recording solution for 30 min at 37°C. H_2_DCFDA is oxidized to the fluorescent dichlorofluorescein (DCF), which is monitored at excitation and emission wavelengths of 488 and 510 nm, respectively, using a TILLvisION digital imaging system. After incubating cells with different reagents, the ROS levels were determined by comparing the changes in fluorescence intensity with that in the standard extracellular recording solution. The fluorescence values were determined by averaging the fluorescence values of at least 50 cells/treatment.

### Detection of mitochondrial membrane potential

The mitochondrial membrane potential was monitored using rhodamine-123 (Rh-123) fluorescent dye imaging. A Raw 264.7 cell suspension was loaded with 10 µg/ml Rh-123 at room temperature for 15 min. After loading, the cells were continuously perfused with recording solution. Rh-123 fluorescence images were captured using the method described above. The fluorescence was excited at 490 nm and filtered at 530 nm. The fluorescence values were determined by averaging the fluorescence values of at least 50 cells/treatment.

### Reagents and solutions

The standard BS comprised the following (in mM): 130 NaCl, 10 CsCl, 1.2 MgCl_2_, 1.5 CaCl_2_, 10 HEPES and 10 glucose titrated to pH 7.4 with NaOH. The BS was then adjusted to an osmotic pressure of 290 mOsm with D-mannitol. The recording BS lacked Ca^2+^. When indicated, 1.5 mM CaCl_2_ was added back to the BS. Stock solutions of glibenclamide, pinacidil, diazoxide, diphenyleneiodonium (DPI), H_2_DCFDA, and Rh-123 were prepared in dimethylsulfoxide (DMSO). 5-hydroxydecanoate (5-HD), tiron, 4-aminopyridine (4-AP), and tetraethylammonium (TEA) were dissolved in distilled H_2_O. All chemicals were obtained from Sigma (St. Louis, MO, USA) and diluted on the day of the experiment.

### Data analysis

The data were expressed as means ± standard errors. The unpaired Student's *t*-test and one-way ANOVA were used for statistical analysis where appropriate. *P*<0.05 was considered statistically significant.

## Results

### Effect of glibenclamide on the resting [Ca^2+^]_i_ of Raw 264.7 macrophages

**Figure 1:**
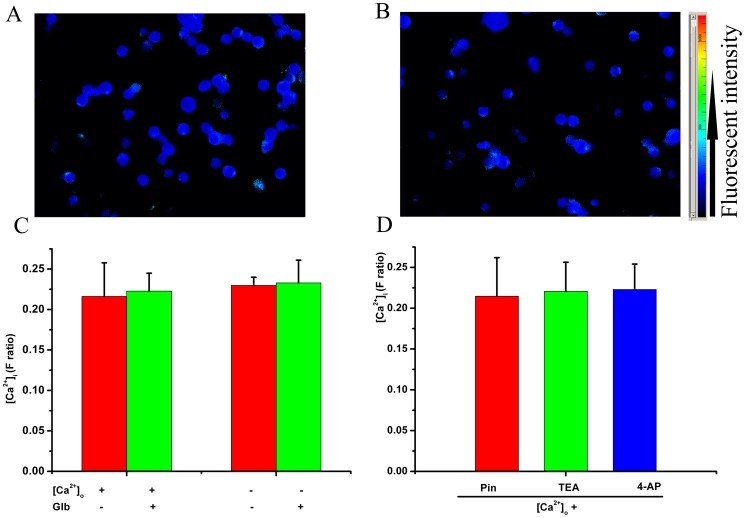
Effect of glibenclamide on the resting [Ca^2+^]_i_ of Raw 264.7 macrophages. A, representative calcium imaging of Raw 264.7 cells in control solution. B, representative calcium imaging with glibenclamide treatment (100 µM). C, glibenclamide has no effect on the resting [Ca^2+^]_i_ with or without calcium in the extracellular solution. D, pinacidil and other unselective potassium channel blockers (4-aminopyridine, 4-AP, 10 µM; tetraethylammonium, TEA, 100 µM) did not change the resting [Ca^2+^]_i_.

Glibenclamide (100 µM) had no effect on the resting [Ca^2+^]_i_ of Raw 264.7 cells with or without Ca^2+^ in extracellular solution (*P*>0.05, Figure 1A, C). Membrane K_ATP_ opener (pinacidil, 100 µM) and other unselective potassium channel blockers (TEA, 100 µM; 4-AP,10 µM) did not change the resting [Ca^2+^]_i_ (*P*>0.05, Figure 1B, D).

### Extracellular ATP-induced [Ca^2+^]_i_ transient elevation

**Figure 2:**
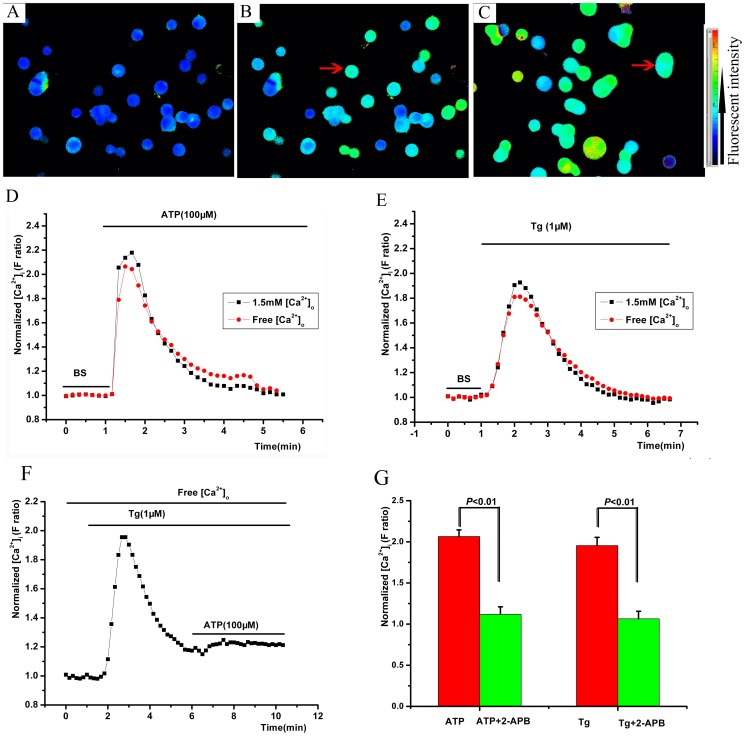
Extracellular ATP induced a [Ca^2+^]_i_ transient elevation in macrophages. A, representative calcium imaging in control solution. B, calcium imaging with ATP (100 µM), the red arrow showed the peak [Ca^2+^]_i_. C, calcium imaging with thapsigargin (Tg, 1 µM), the red arrow showed the peak [Ca^2+^]_i_. D, time series of the ratio of fluorescence intensity at excitation wavelengths of 340 and 380 nm (F ratio) during the application of ATP. E, time series of the mean F ratio during the application of Tg. F, time series of the mean F ratio during the sequential application of Tg and ATP. G, 2-aminoethoxydiphenyl borate (2-APB, 100 µM) inhibited the [Ca^2+^]_i_ transient elevation induced by both ATP and Tg. BS, bath solution.

Extracellular ATP (100 µM) induced [Ca^2+^]_i_ transient elevation in Raw 264.7 cells (Figure 2A, B, D). The transient elevation was independent of the extracellular Ca^2+^ (*P*<0.05, Figure 2D). Thapsigargin (Tg, 1 µM) also induced [Ca^2+^]_i_ transient elevation in Raw 264.7 cells (Figure 2 E) in the absence and presence of extracellular Ca^2+^. When the intracellular calcium stores were released by Tg (1 µM), ATP could not induce [Ca^2+^]_i_ transient elevation again (Figure *P*<0.05, 2C, F). 2-APB (100 µM) was able to block the [Ca^2+^]_i_ transient elevation induced by both ATP and Tg (*P*<0.05, Figure 2F). Moreover, extracellular ATP (100 µM) induced [Ca^2+^]_i_ transient in THP cells (Figure S1), independent of the extracellular Ca^2+^.

When Raw 264.7 cells were treated with ATP (100 µM) or Tg (1 µM) in Ca^2+^-free buffer and then perfused with 1.5 mM Ca^2+^ extracellular buffer, [Ca^2+^]_i_ was very modestly increased, which then completely recovered to baseline levels quickly (Figure S2A, B).

### Effect of glibenclamide on ATP-induced [Ca^2+^]_i_ transient elevation

**Figure 3:**
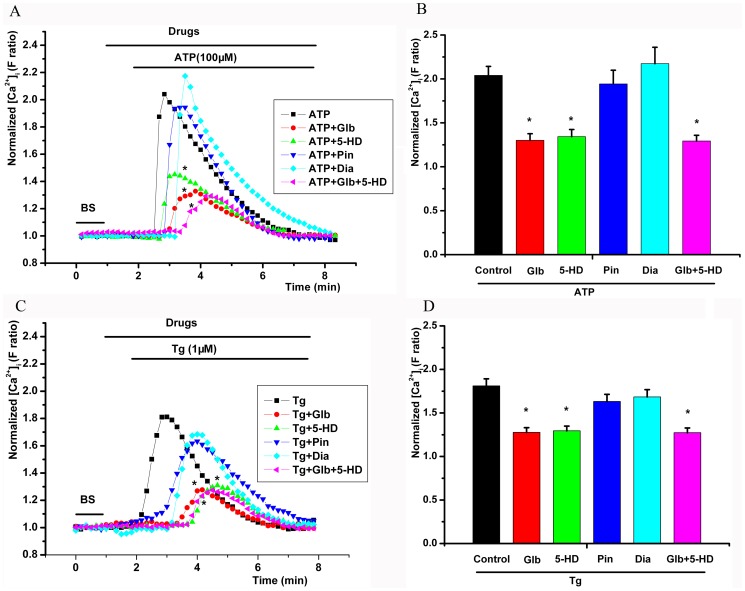
Effect of glibenclamide on the [Ca^2+^]_i_ transient elevation induced by extracellular ATP. A, C, time series of the mean F ratio during the application of different agents. B, D, the maximal [Ca^2+^]_i_ with different agents. Glibenclamide (100 µM) and 5-hydroxydecanoate (5-HD, 100 µM) decreased the ATP- or Tg-induced peak [Ca^2+^]_i_; pinacidil (100 µM) did not change the maximal [Ca^2+^]_i_; diazoxide (Dia, 100 µM) increased the peak [Ca^2+^]_i_ without significant differences. * *P*<0.05, compared with the control. BS, bath solution.

Glibenclamide (100 µM) decreased the peak of the [Ca^2+^]_i_ transient elevation induced by extracellular ATP (100 µM) (*P*<0.05, Figure 3 A, B). Pinacidil (100 µM) and other unselective potassium channel blockers (TEA and 4-AP) did not change the maximal [Ca^2+^]_i_ (Figure 3 A, B). The mitochondrial K_ATP_ blocker 5-HD (100 µM) also decreased the maximal [Ca^2+^]_i_ (*P*<0.05, Figure 3 A, B). When simultaneously pretreated with glibenclamide (100 µM) and 5-HD (100 µM), the maximal [Ca^2+^]_i_ transient was decreased to the same level as that by either glibenclamide or 5-HD alone (*P*>0.05, Figure 3 A, B).

Glibenclamide (100 µM) or 5-HD (100 µM) also decreased the peak of the [Ca^2+^]_i_ transient elevation induced by extracellular Tg (1 µM) (*P*<0.05, Figure C, D).

### Effect of ROS inhibitors on ATP-induced [Ca^2+^]_i_ transient elevation

**Figure 4:**
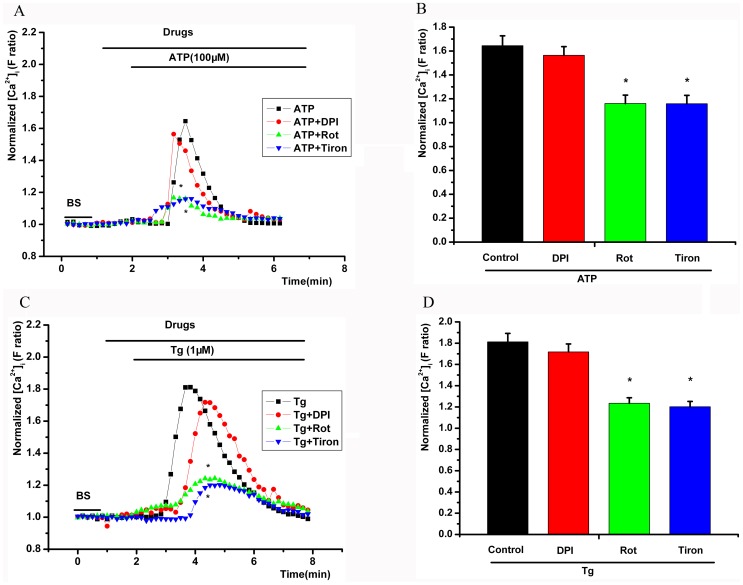
Effect of various intracellular ROS inhibitors on the [Ca^2+^]_i_ transient elevation induced by extracellular ATP. A, C, time series of the mean F ratio during the application of different agents. B, D, the maximal [Ca^2+^]_i_ with different agents. The NADPH oxidase inhibitor diphenyleneiodonium (DPI, 10 µM) did not decrease the ATP- or Tg-induced maximal [Ca^2+^]_i_. However, the ROS scavenger tiron (1 mM) and mitochondrial inhibitor rotenone (Rot, 5 µM) inhibited the extracellular ATP- and Tg-induced maximal [Ca^2+^]_i_. * *P*<0.05, compared with the control.

The NADPH oxidase (NOX) inhibitor DPI (10 µM) did not decrease the maximal [Ca^2+^]_i_ induced by extracellular ATP (*P*>0.05, Figure 4 A, B). However, an ROS scavenger (tiron, 1 mM) and a mitochondria inhibitor (rotenone, 5 µM) inhibited the extracellular ATP-induced maximal [Ca^2+^]_i_ (*P*<0.05, Figure 4 A, B). DPI had no effect on the maximal [Ca^2+^]_i_ induced by extracellular Tg (*P*>0.05, Figure 4 C, D), but tiron and rotenone decreased Tg-induced maximal [Ca^2+^]_i_ (*P*<0.05, Figure 4 C, D).

### Effect of glibenclamide on intracellular ROS and mitochondrial activity

**Figure 5:**
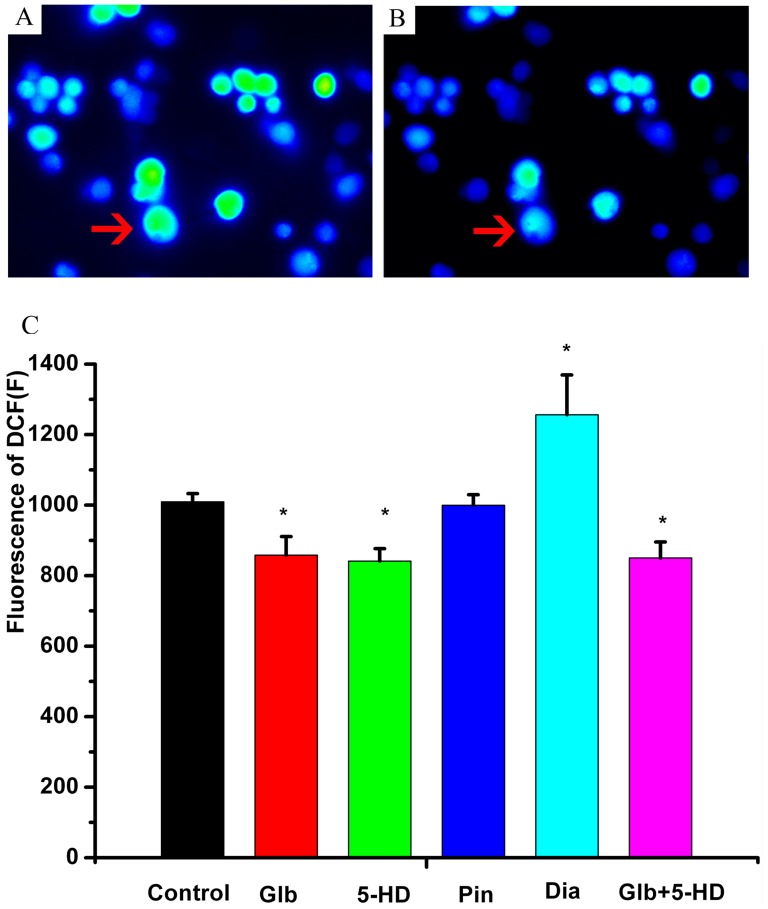
Effect of glibenclamide on intracellular ROS. A, representative intracellular ROS imaging in the control solution, the red arrow showed the fluorescence of dichlorofluorescein (DCF). B, representative ROS imaging with glibenclamide (100 µM). C, statistics of intracellular ROS levels with different agents. * *P*<0.05, compared with the control.

**Figure 6:**
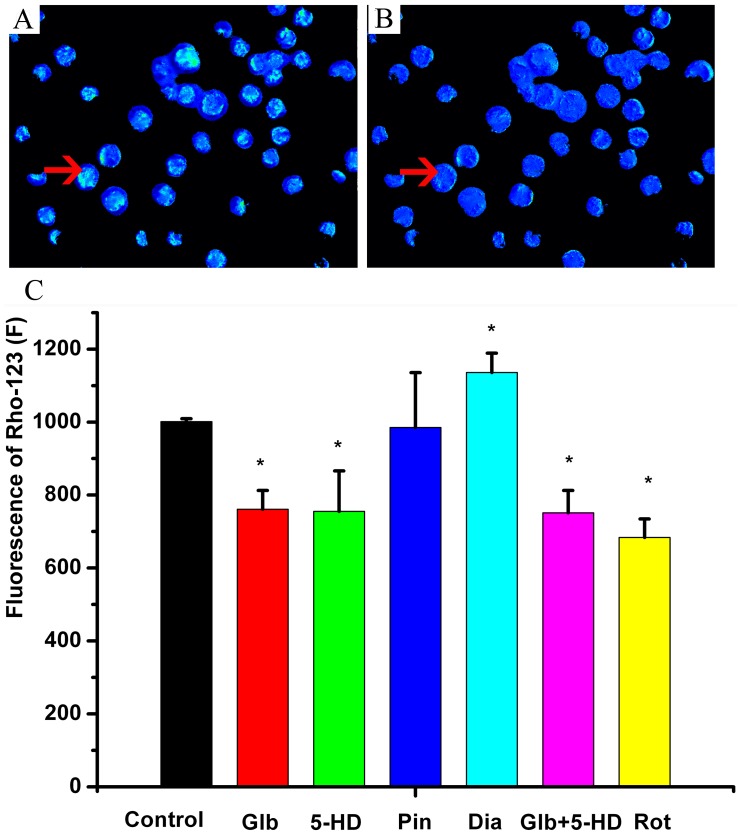
Effect of glibenclamide on mitochondrial activity. A, representative mitochondrial activity imaging in the control solution, the arrow showed the intensity of rhodamine-123 (Rh-123). B, representative mitochondrial imaging with glibenclamide (100 µM). C, Statistics of the intensity of Rh-123 using different agents. * *P*<0.05, compared with the control.

Glibenclamide or 5-HD could decrease the level of intracellular ROS detected based on the fluorescence of DCF (*P*<0.05, Figure 5). Pinacidil and other unselective potassium channel blockers (TEA and 4-AP) did not change intracellular ROS (*P*>0.05, Figure 5). Glibenclamide or 5-HD also decreased the fluorescence intensity of Rh-123, reflecting the depolarization of the mitochondrial membrane potential (*P*<0.05, Figure 6). Diazoxide could increase the intensity of Rh-123 and intracellular ROS (*P*<0.05, Figure 6). When simultaneously pretreated with glibenclamide (100 µM) and 5-HD (100 µM), the fluorescence intensity of DCF (*P*>0.05, Figure 5) and Rh-123 (*P*>0.05, Figure 6) was decreased to the same level as that individually decreased by glibenclamide or 5-HD.

### The role of intracellular ROS and mitochondrial activity in the effect of glibenclamide on the ATP-induced [Ca^2+^]_i_ transient elevation

**Figure 7:**
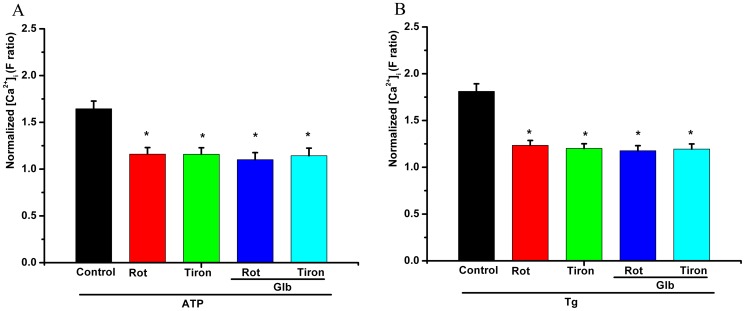
The role of intracellular ROS and mitochondrial activity in the effect of glibenclamide on ATP-induced [Ca^2+^]_i_ transient elevation. When pretreated with tiron and rotenone, glibenclamide could not further decrease the maximal ATP- or Tg-induced [Ca^2+^]_i_.

When pretreated with tiron and rotenone, glibenclamide and 5-HD could not further decrease the ATP (100 µM)- or Tg (1 µM)-induced maximal [Ca^2+^]_i_ transient elevation (*P*>0.05, Figure 7).

## Discussion

In the present study, for the first time, we have demonstrated the following: 1) glibenclamide cannot affect the resting [Ca^2+^]_i_ of Raw 264.7 macrophages; 2) extracellular ATP induced the [Ca^2+^]_i_ transient elevation in Raw 264.7 macrophages by depleting [Ca^2+^]_i_ stores; 3) intracellular ROS can regulate the ATP-induced [Ca^2+^]_i_ transient; and 4) glibenclamide decreased the ATP-induced [Ca^2+^]_i_ transient elevation by inhibiting ROS that were mainly generated from the mitochondria in macrophages.

Cytosolic Ca^2+^ was considered to be an important second messenger to activate immune cells [6], [7]. Resting lymphocytes were found to maintain a low concentration of [Ca^2+^]_i_ [6]. Cytosolic Ca^2+^ elevations are required for diverse immune cellular functions, including differentiation, effector function, and gene transcription [7], [17]. Recently, we found that glibenclamide could ameliorate the progression of atherosclerosis and reduce the production of inflammatory cytokines as well as the phosphorylation of p65 and ERK1/2 [5]. However, it is unclear that glibenclamide can affect [Ca^2+^]_i_ in macrophages. In the present study, we found that glibenclamide did not affect the resting [Ca^2+^]_i_ of Raw 264.7 macrophages, which was very different than the effect of glibenclamide on excitable cells, such as smooth muscle cells [18], neurons [19], and β-cells [2]. In these cells, glibenclamide inhibits membrane K_ATP_, leading to membrane depolarization, which in turn opens voltage-gated calcium channels and increases [Ca^2+^]_i_ via Ca^2+^ influx.

At sites of inflammation, macrophages are exposed to various chemical mediators, such as nucleotides, prostanoids, and oxygen radicals [8], [9]. In particular, increasing evidence has suggested that extracellular ATP participates in the inflammatory response as a proinflammatory mediator through its capacity to bind and activate multiple nucleotide receptor family members [10]. In non-excitable cells, extracellular ATP induces an elevation of cytosolic Ca^2+^. In the present study, we found that ATP induced [Ca^2+^]_i_ transient elevation independent of extracellular Ca^2+^ in Raw 264.7 cells. We also found this phenomena in THP-1 cells, whereas other studies found that ATP induced [Ca^2+^]_i_ independent of extracellular Ca^2+^ in Sertoli cells [20], the principal cells of the inner medullary collecting duct [21], and human monocytes [22]. Thus, ATP induced [Ca^2+^]_i_ transient independent of extracellular Ca^2+^ in a manner that was not specific to macrophages.

When the calcium stores were pre-released by thapsigargin, ATP could not induce [Ca^2+^]_i_ transient again. The Ca^2+^ influx was very modest and recovered to the baseline level quickly when 1.5 mM Ca^2+^ was reintroduced to the extracellular buffer following pretreatment with ATP or Tg in Ca^2+^-free buffer. Mikulski Z *et al* [23] also found that nicotinic receptors on rat alveolar macrophages dampened the ATP-induced Ca^2+^-release from intracellular stores. In murine J774 macrophages, low concentrations (10 µM) of ATP evoked Ca^2+^ transient in a phospholipase C (PLC)-dependent manner [24]. These results suggested that the Ca^2+^ influx resulting from the depletion of Ca^2+^ stores was modest and transient in Raw 264.7 cells and was covered by ATP- or Tg-induced [Ca^2+^]_i_ release. Furthermore, we found that 2-APB, a membrane permeable IP_3_ receptor antagonist [25], was able to inhibit the [Ca^2+^]_i_ elevation induced by ATP. Many studies found that ATP induced Ca^2+^ release from the endoplasmic reticulum (ER) through inositol 1,4,5- trisphosphate (IP_3_) receptors (IP_3_Rs) [26], [27], and these results suggested that ATP induced the [Ca^2+^]_i_ transient elevation by releasing Ca^2+^ from the intracellular Ca^2+^ stores in Raw 264.7 macrophages.

Because previous studies showed that glibenclamide has a wide range of anti-inflammatory effects [3], [4], investigating whether glibenclamide produces any effect on the ATP-induced [Ca^2+^]_i_ transient elevation is worthwhile. For the first time, we found that glibenclamide decreased the maximal [Ca^2+^]_i_ transient elevation induced by extracellular ATP or Tg. However, a membrane K_ATP_ opener (pinacidil) and other unselective potassium channel blockers (TEA and 4-AP) did not change the maximal [Ca^2+^]_i_ transient. The mitochondrial K_ATP_ blocker (5-HD) also decreased the maximal [Ca^2+^]_i_ transient. Glibenclamide and 5-HD showed no additive effect on decreasing the maximal [Ca^2+^]_i_ transient. Because glibenclamide has been shown to have an inhibitory effect on mitochondria K_ATP_ channels [28], the results suggested that glibenclamide might inhibit ATP-induced [Ca^2+^]_i_ transient elevation by blocking mitochondrial K_ATP_ channels.

However, the link between mitochondrial K_ATP_ channels and ATP-induced intracellular calcium transient is not clear. Recently, Hänninen SL *et al* [29] found that mitochondrial uncoupling downregulated calsequestrin expression and reduced sarcoplasmic reticulum Ca^2+^ stores in cardiomyocytes. Thus, we hypothesized that ROS mainly from the mitochondria participated in the regulation of ATP-induced [Ca^2+^]_i_ transient and that glibenclamide might inhibit this transient by inhibiting ROS generation by blocking mitochondrial K_ATP_ channels.

First, we used different ROS inhibitors to detect which sources of ROS affected the ATP-induced [Ca^2+^]_i_ transient elevation. The NOX inhibitor DPI did not decrease the maximal [Ca^2+^]_i_ transient induced by extracellular ATP or Tg. An ROS scavenger (tiron) and a mitochondrial inhibitor (rotenone) inhibited the extracellular ATP- or Tg- induced maximal [Ca^2+^]_i_. Mitochondria depolarization was previously found to increase the generation of mitochondria-derived ROS, which stimulated Ca^2+^ sparks in cerebral artery smooth muscle cells [30]. Rakesh Rathorea *et al* [31] found that hypoxia might specifically increase mitochondrial ROS generation, which subsequently contributed to a hypoxia-induced increase in [Ca^2+^]_i_ and contraction in the pulmonary artery smooth muscle cells. These results suggested that ROS mainly from the mitochondria participated in the regulation of the ATP-induced intracellular calcium in Raw 264.7 macrophages.

Second, we explored whether glibenclamide could decrease the level of intracellular ROS. Glibenclamide or 5-HD could decrease the level of intracellular ROS, but pinacidil and other unselective potassium channel blockers (TEA and 4-AP) did not change the intracellular ROS. Glibenclamide and 5-HD also decreased the intensity of Rh-123 staining, reflecting depolarization of the mitochondrial membrane potential. Diazoxide was able to increase the intensity of Rh-123 staining and the intracellular ROS level. Glibenclamide and 5-HD showed no additive effect in decreasing the fluorescence intensity of DCF and Rh-123. Thus, glibenclamide could be concluded to decrease the level of intracellular ROS by inhibiting mitochondrial activity and blocking mitochondrial K_ATP_ channels.

Finally, when pretreated with tiron and rotenone, glibenclamide and 5-HD could not further decrease the maximal ATP- or Tg-induced [Ca^2+^]_i_ transient. Additionally, glibenclamide had no effect on [Ca^2+^]_i_ transient in Raw 264.7 macrophages pretreated with 5-HD. Because mitochondrial K_ATP_ channels play an important role in ROS production [15], we could conclude that the inhibitory effect of glibenclamide on the ATP-induced [Ca^2+^]_i_ transient elevation might be mediated by inhibiting ROS and blocking mitochondrial K_ATP_ channels.

In conclusion, extracellular ATP induced the intracellular calcium transient elevation by depleting calcium stores in Raw 264.7 macrophages, which could be regulated by ROS mainly from mitochondria. Glibenclamide might inhibit this transient activity by blocking mitochondrial K_ATP_ channels, resulting in the inhibition of the cross-talk among [Ca^2+^]_i_, intracellular ROS, and mitochondrial activity. However, further studies should be performed to reveal the effect of glibenclamide on other physiological functions of macrophage cells by regulating [Ca^2+^]_i_ homeostasis.

## Supporting Information

Figure S1 **Extracellular ATP induced a [Ca^2+^]_i_ transient elevation in THP-1 cells.** Time series of the ratio of the fluorescence intensity at excitation wavelengths of 340 and 380 nm (F ratio) during the application of ATP (100 µM) in THP-1 cells. BS, bath solution.
(TIF)

Figure S2 **Store-operated calcium influx induced by ATP and thapsigargin.** When Raw 264.7 cells were treated with ATP (A, 100 µM) or Tg (B, 1 µM) in Ca^2+^-free buffer and then perfused with 1.5 mM Ca^2+^ extracellular buffer, the [Ca^2+^]_i_ was modestly increased and then completely recovered to the baseline levels quickly.
(TIF)
