# Supplementary figures and images for: Glibenclamide Decreases ATP-Induced Intracellular Calcium Transient Elevation via Inhibiting Reactive Oxygen Species and Mitochondrial Activity in Macrophages
Source: PLoS One. 2014 Feb 18;9(2):e89083. doi: 10.1371/journal.pone.0089083 (PMC3928368; doi:10.1371/journal.pone.0089083)

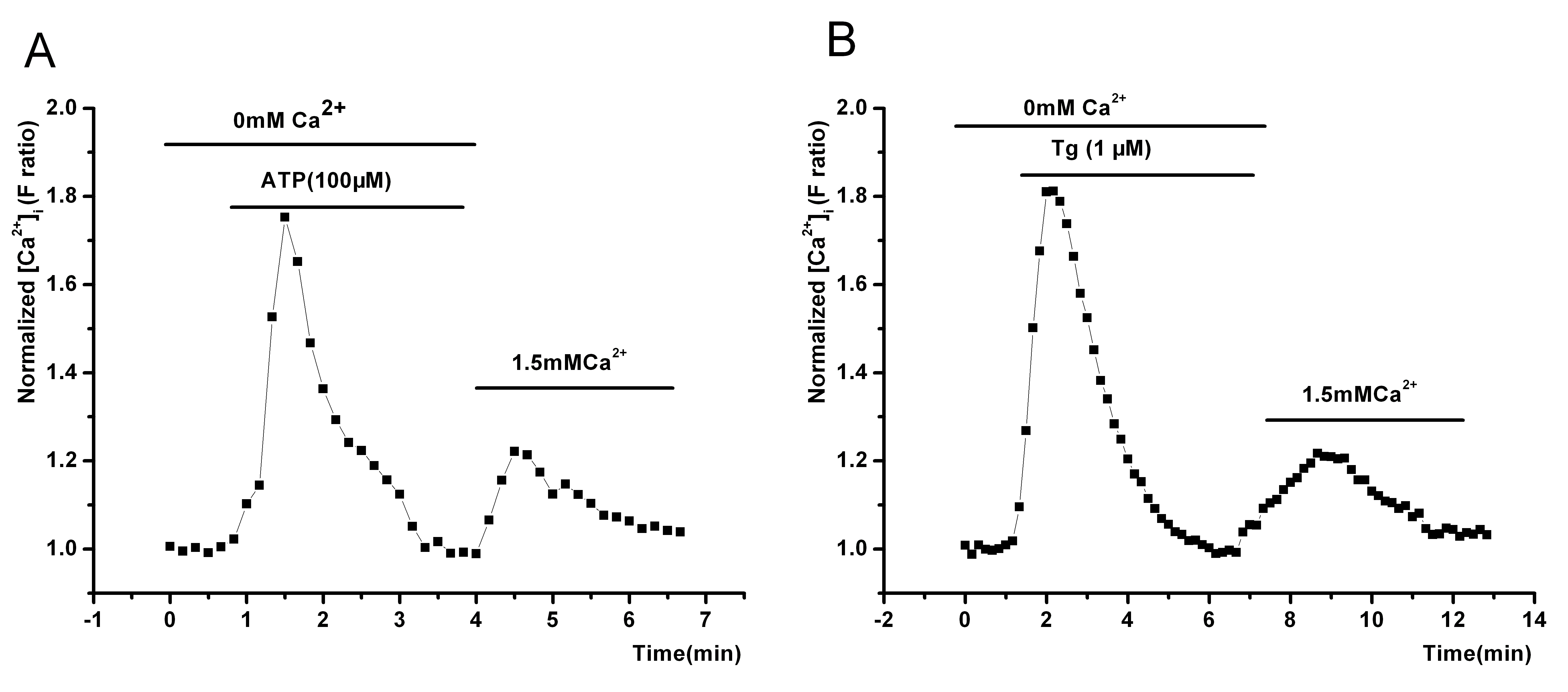

Supplement: Figure S2 — Store-operated calcium influx induced by ATP and thapsigargin. When Raw 264.7 cells were treated with ATP (A, 100 µM) or Tg (B, 1 µM) in Ca2+-free buffer and then perfused with 1.5 mM Ca2+ extracellular buffer, the [Ca2+]i was modestly increased and then completely recovered to the baseline levels quickly. (TIF) [file pone.0089083.s002.tif]
